# Motion information plays only a secondary role in sex identification of walking persons in frontal view

**DOI:** 10.1167/jov.23.2.11

**Published:** 2023-02-22

**Authors:** Maarten F. Bobbert, Marit V. Lemmens, Melanie J. Groeneveld

**Affiliations:** 1 Faculty of Behavioural and Movement Sciences, Department of Human Movement Sciences, Vrije Universiteit Amsterdam, Amsterdam Movement Sciences, Amsterdam, The Netherlands; 2 Faculty of Behavioural and Movement Sciences, Department of Human Movement Sciences, Vrije Universiteit Amsterdam, Amsterdam, The Netherlands; 3 Faculty of Behavioural and Movement Sciences, Department of Human Movement Sciences, Vrije Universiteit Amsterdam, Amsterdam, The Netherlands

**Keywords:** point-light walker, image, movie, gender recognition, gender discrimination

## Abstract

Observers have a success rate above chance in identifying the sex of walking persons on the basis of movies showing only point lights. It has been claimed that observers rely heavily on motion information for their judgment. Here, we studied, for the frontal plane, the added value of motion information over just form information. In the first experiment, we asked 209 observers to identify the sex of frontal-plane still images of point lights of six male and six female walkers. We used two types of point-light images: (1) cloud-like images, showing just point lights, and (2) skeleton-like images with point lights interconnected. On the basis of cloud-like still images, observers had a mean success rate of 63%; on the basis of skeleton-like still images, they had a higher mean success rate of 70% (*p* < 0.001). In the second experiment, we asked 273 observers to identify the sex of skeleton-like still images and skeleton-like movies of eight full walking strides. The overall success rate based on movies was 73%. Among the observers first presented with still images, the success rate based on still images was 68%, but for observers first presented with movies the success rate based on still images was 74%, not different from that based on movies (*p* > 0.05). Our interpretation was that motion information revealed what the point lights represented but had no additional value when this became clear. Hence, we concluded that motion information plays only a secondary role in sex identification of walking persons in the frontal plane.

## Introduction

Imagine the following. You see a person walking at a distance; the distance is too great to see what kind of clothes the person is wearing or what the person's hair looks like. Can you identify whether this person is a man or a woman? We all have the feeling that we are able to do so with a success rate above chance, even in adverse lighting conditions. This feeling is corroborated by the results of scientific studies. In most of these studies, point-light images of walking people have been used. Such images, first introduced by Johansson (1973), only show point lights attached to the body at joints of the upper and lower extremities; the body itself is invisible, and hence there are no obvious sex cues such as clothing style and hairstyle. Still images of a walking cycle can be shown, or images can be shown in succession to create a movie. According to a meta-analysis (Pollick, Kay, Heim, & Stringer, 2005), observers had a success rate of 66% in identifying the sex of walkers from point-light movies in a sagittal view; in frontal and oblique views, the success rate was 71%. What types of information are observers using to decide the sex of a walker from point-light movies?

According to the literature (e.g., Troje, 2002), there are two types of information that an observer can extract from a point-light movie: (1) structural information, also referred to as “form”; and (2) dynamic information, also referred to as “motion.” Structural information includes information about body size and shape, and dynamic information includes the precise trajectories and velocities of body parts. In terms of structure, men are on average taller and have greater absolute shoulder width (e.g., Barclay, Cutting, & Kozlowski, 1978; Mather & Murdoch, 1994); in relative terms, they have a greater ratio of shoulder width to hip width (e.g., Barclay et al., 1978; Hughes & Gallup, 2003; Lippa, 1983; Mather & Murdoch, 1994). In terms of gait pattern, men make on average longer strides at a lower cadence than women (e.g., Bruening, Frimenko, Goodyear, Bowden, & Fullenkamp, 2015), and it has been argued that men have more lateral body sway in shoulders and less lateral sway in hips than women (Mather & Murdoch, 1994). Furthermore, according to Hiris, Mirenzi, and Janis (2016), males keep their elbows farther away from the body and have greater distances between their knees and ankles during walking than females.

A fundamental question raised in the literature is to what extent observers use structural information and to what extent they use dynamic information on the gait pattern when trying to identify the sex of a walking person. Considering that a still image contains structural information but no dynamic information, whereas a movie contains both types of information, a straightforward approach to finding an answer to the question is to compare the success rate of sex identification on the basis of still images with that on the basis of movies. This approach was taken by Kozlowski and Cutting (1977). They showed observers both point-light images and point-light movies in the sagittal plane. Looking at a collage of four still images extracted from the gait cycle, their observers did not perform above chance in identifying the sex of the walkers; the success rate was 46%. Looking at the movies, however, the observers had a success rate of 63%, statistically significantly above chance. Based on these results, Kozlowski and Cutting (1977) concluded that observers need dynamic information to identify the sex of a sagittal-plane point-light display of a walking person.

It may be argued that, in the sagittal plane, structural information is not very helpful because most of the anthropometric differences between men and women are difficult to observe in that plane (Cutting, Proffitt, & Kozlowski, 1978). Mather and Murdoch (1994) synthesized frontal-plane point-light movies that had either a male or a female torso structure, each with either male or female body sway, and presented these to observers. The authors concluded that, for dynamic frontal projections, sex identification is mediated by lateral body sway in shoulders and hips and depends on dynamic motion cues rather than structural information. Troje (2002) concluded for frontal-plane point-light movies that dynamic information was about equally important as structural information. Davis and Gao (2004) showed observers both point-light images and point-light movies in the frontal plane. Looking at still images, their observers had a success rate of 57%. Looking at point-light movies, observers had a substantially higher success rate of 69%. Hence, the authors concluded that, in the frontal plane, point-light movies give more sex-related information than point-light still images, in line with the conclusion drawn by Kozlowski and Cutting (1977) for the sagittal plane.

The consensus of opinion among the experts seems to be, then, that observers rely heavily on dynamic information when trying to identify the sex of walking persons. It has been suggested, however, that the importance of dynamic information may depend on the amount of ambiguity present in the stimulus (Lu, 2010). This brings us to the question of to what extent the outcome depends on the properties of the point-light images and movies used. Kozlowski and Cutting's point-light images were high-contrast video images of persons walking with 5-cm wide retroreflective tape wrapped around parts of their bodies. The markers were large (due to videotape dubbing, they even “bloomed” slightly) and were presented disconnected from each other in a cloud, and some markers could be occluded in the sagittal view by intervening body parts. The authors reported that when first presented with the point-light images, only one of their 20 observers guessed that they represented people. It is not surprising, then, that the observers could not identify the sex of the persons! The limitations of traditional movie capture can be easily overcome by modern motion capture technology. Nowadays, it is easy to make high-resolution point-light images and movies with many markers that can be presented as small dots rather than big blobs so that anatomical details are preserved. Davis and Gao (2004) presented frontal-plane point-light still images with 13 markers, disconnected from each other in a cloud. Before the start of their experiment, they included a preview stage in which the observers were shown a point-light movie and were told that it contained a person walking on a treadmill with markers attached. It was after this preview stage that the observers had a success rate of 57% in identifying the sex on the basis of point-light still images. This success rate was above chance, but still low. Is this the best observers can do on the basis of point-light still images? Why not present the observers with point-light still images in which the markers are interconnected with lines so that body segments are visible, which makes it immediately clear that the images represent people? Interconnecting the markers by lines to create stick diagrams has been done in the study of sex identification on the basis of point-light movies (e.g., Troje, 2002), but has not been used in the study of sex identification on the basis of point-light still images as far as we know. We feel that in order to study the relative importance of structural and dynamic information in identifying the sex on the basis of point-light images and movies, the structural information should be presented as clearly as possible. It should be clear to the observer that he or she is looking at a human body; the question at issue is to what extent the identification of the sex of this body additionally requires dynamic information.

**Figure 1.**
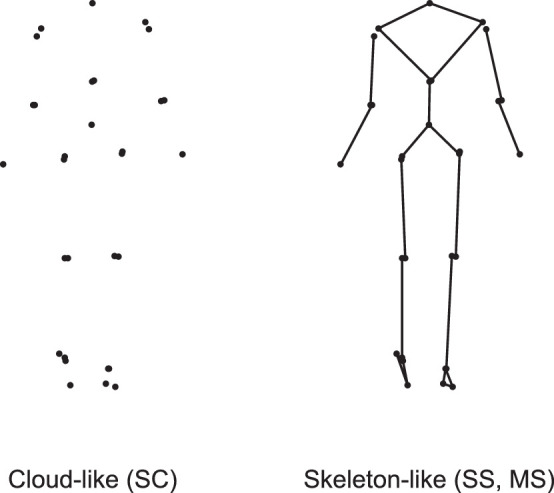
A point-light image can be constructed with marker dots disconnected in a cloud or with marker dots interconnected by lines to create a kind of skeleton. In this study, we used still images that were cloud like (SC, left), and still images and movies that were skeleton like (SS and MS, right). SC and SS images were extracted from MS videos at the instant that the right heel contacted the ground. We displayed the following virtual joints calculated from cluster markers: shoulder joints, elbow joints, wrist joints, C7–T1, T12–L1, L5–S1, hip joints, knee joints, and ankle joints. Also, markers at the heels and big toes were displayed. Because the locations of some virtual joints could be calculated independently from two clusters of markers, some segments may seem “dislocated.” For details, see van Leeuwen et al. (2020a). The observers had to answer the question: “Is this a man or a woman?”

The purpose of the present study was to determine to what extent observers need dynamic information to identify the sex of a walking person in the frontal plane. Because the answer might depend on how the point lights are presented, we tested in the first experiment whether observers perform better on the basis of point-light still images that are skeleton like, containing marker dots interconnected by lines (Figure 1, right), rather than cloud like, containing only marker dots (Figure 1, left). In the second experiment, we addressed the additional value of dynamic information by testing whether observers perform better on the basis of skeleton-like point-light movies than on the basis of skeleton-like point-light still images. We chose to use skeleton-like images and movies in this second experiment because it is more ecologically valid; after all, in real life we try to identify moving bodies rather than disconnected points floating in space.

## Experiment 1: Sex identification of cloud-like and skeleton-like still images

### Methods

#### Design

**Figure 2.**
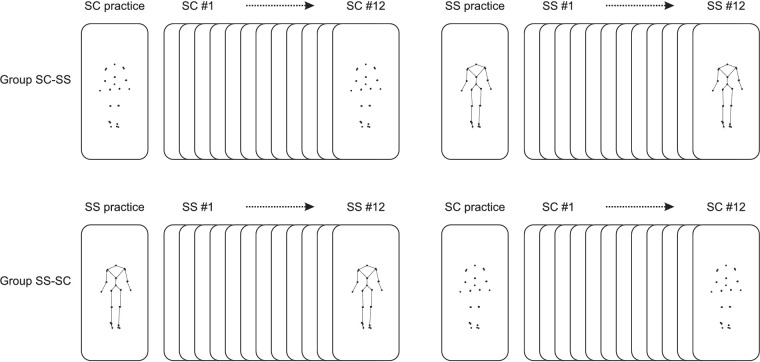
Design of Experiment 1. Each observer was randomly assigned to group SC–SS or group SS–SC. Observers in group SC–SS first saw 13 SC images (still images that were cloud like) and then 13 SS images (still images that were skeleton like). Observers in group SS–SC first saw 13 SS images and then 13 SC images. The first image in a block was not included in the analysis; its purpose was to get an impression of what the observer saw when he or she was still naïve (the question was posed: “What do you see?”) and give an example of the images and question that would follow in the block.

In this experiment, observers tried to identify the sex of point-light still images in the frontal plane created from kinematic data of six walking females and six walking males. Two types of still images were shown: still cloud-like (SC; Figure 1, left) and still skeleton-like (SS; Figure 1, right). After the observers had studied an image, they answered the question: “Is this a man or a woman?” The observers did not receive any feedback on their answers and hence had no idea as to whether they had any success. We used a crossover design. In random order, half of the observers (group SC–SS) were first presented with a block of SC images and then with a block of SS images, and the other half (group SS–SC) were first presented with a block of SS images and then with a block of SC images (Figure 2). We asked the following specific questions:

1. Are observers performing above chance when trying to identify the sex of the walkers on the basis of SC images and on the basis of SS images?
2. Are observers more successful in identifying the sex of the walker on the basis of SS images than on the basis of SC images?
3. Are observers more successful in identifying the sex of the walkers on the basis of SC images if they have experience with identifying the sex on the basis of SS images?

The experiment was designed in accordance with the code of ethics for research in the social and behavioral sciences involving human participants as accepted by the Deans of Social Sciences in the Netherlands in 2016 and was approved by the local ethics committee.

#### Construction of images

Frontal-plane point-light images at the instant that the right heel contacted the ground (Figure 1) were constructed from 50-Hz three-dimensional (3D) kinematics of marker clusters applied to persons walking on a treadmill at normalized speed (1.25 m/s times the square root of leg length in meters); these kinematics had been collected as part of an earlier, independent study by fellow researchers (van Leeuwen, van Dieën, Daffertshofer, & Bruijn, 2020a; van Leeuwen, van Dieën, Daffertshofer, & Bruijn, 2020b). From their data on 30 subjects, we selected the data of six males and six females, matched in walking speed and cadence (Table 1) to neutralize sex cues in gait parameters of men and women (e.g., Bruening et al., 2015). For these subjects, we constructed point-light cloud-like and skeleton-like movies, from which we extracted still images at right heel strike to obtain SC and SS images, respectively.

**Table 1.** Anthropometrics and gait parameters of six male and six female walking subjects whose kinematic data were used to create point-light images and movies.

	Mean (SD)				
	Age (yr)	Mass (kg)	Stature (m)	Walking speed (m/s)	Cadence (steps/min)
Males	35 (12)	73 (8)	1.80 (0.07)	1.20 (0.04)	135 (7)
Females	30 (6)	63 (5)	1.69 (0.08)	1.20 (0.04)	134 (3)

#### Questionnaire and task of the observers

Using Qualtrics XM (Qualtrics LLC, Seattle, WA), a questionnaire was designed and presented to observers. The procedure of sex identification was as follows: An image was presented (comprising about half of the height of the display used by the observer), and the observer indicated whether in his or her opinion the sex of the walker was male or female. Observers were randomly assigned to group SC–SS or group SS–SC. Observers in group SC–SS first saw 13 SC images and then 13 SS images; observers in group SS–SC first saw 13 SS images and then 13 SC images (Figure 2). The response to the first image in a block of 13 was not included in the analysis; the first image, which was one of the remaining 12 images, was shown to provide the observer with an example of the images and question that would follow in the block and for us to get an impression of what the observer saw when he or she was still naïve (the question was posed: “What do you see?”). To prevent a possible bias due to using a fixed presentation order (Efird, 2011), the 12 SC images following the example SC image were presented in random order, and the same was true for the remaining 12 SS images following the example SS image. The sex selected by the observers for each of these 24 images was used for further analysis. In the questionnaire, we also posed a few other questions to the observers—for example, “Have you watched the images carefully and answered the man/woman question to the best of your ability?” and “What helped you decide whether the image was a man or a woman?”

#### Observers

Observers were recruited among family, friends, acquaintances, and students of the university via WhatsApp, e-mail, Facebook, and Twitter. Of 354 observers who started the questionnaire, 137 did not complete it and were excluded. An additional eight observers were excluded because they had answered “no” to the question of whether they had watched the images carefully. This left us with 209 observers (79 men and 130 women), 93 in group SC–SS and 116 in group SS–SC.

#### Statistical analysis

To determine whether observers performed above chance in identifying the sex on the basis of SC and SS, we used a one-sample Wilcoxon signed-rank test. To answer the question of whether observers were more successful in identifying the sex of SS images than in identifying that of SC images, we used a Wilcoxon signed-rank test for paired data. To answer the question of whether observers were more successful in identifying the sex of SC images if they had experience with identifying the sex of SS images, we used a Wilcoxon rank-sum test on scores of group SC–SS and group SS–SC. All analyses were conducted in MATLAB (MathWorks, Natick, MA), and the level chosen for statistical significance was 5%. Every *Z*-statistic reported in this manuscript is from the Wilcoxon tests.

### Results of Experiment 1

**Figure 3.**
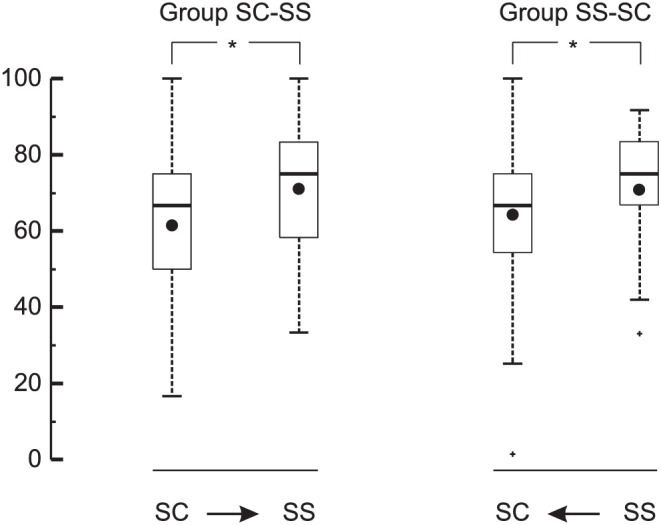
Box plots of success percentages achieved by the two groups in Experiment 1. The central horizontal line in each box indicates the median, and the bottom and top edges of the box indicate the 25th and 75th percentiles, respectively. The whiskers extend to the most extreme data points not considered outliers, and the outliers are plotted individually using plus (+) signs. The black circle in each box indicates the mean value. The arrows indicate the order of presentation for easy reference. Each asterisk (*) signals that the indicated difference was statistically significant (*p* < 0.05).

Box plots of success rates of the two groups are shown in Figure 3. From the whiskers, it can be derived that some observers performed below chance, but statistically speaking the groups performed above chance in identifying the sex of SC images (overall mean 63% correct; *Z* = 8.29; *p* < 0.001) and SS images (overall mean 70% correct; *Z* = 11.73; *p* < 0.001). Observers in both groups were more successful in identifying the sex on the basis of SS images than on the basis of SC images (group SC–SS: *Z* = 3.72, *p* < 0.001; group SS–SC: *Z* = 3.05, *p* < 0.001). First identifying SS images had no effect on the success rate of identifying SC images (*Z* = 1.05; *p* = 0.29).

**Figure 4.**
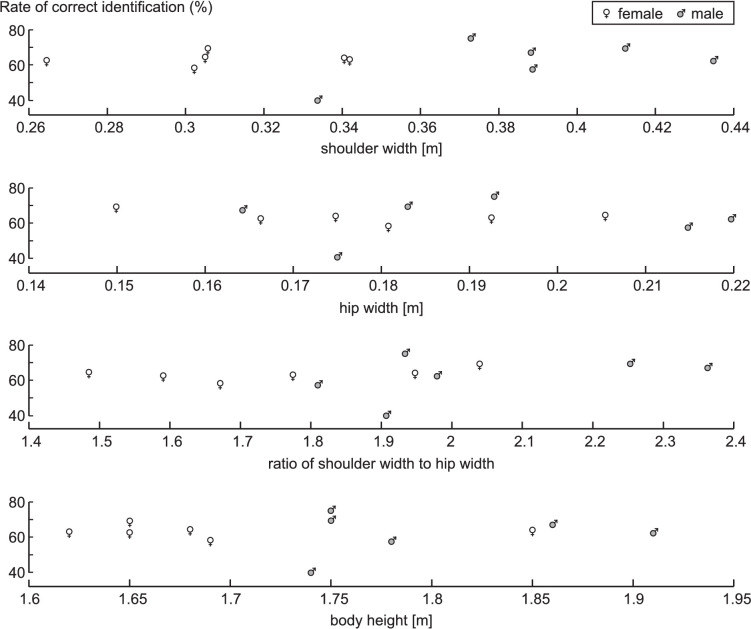
Rate of correct sex identification of the SS images of individual males and females, plotted as a function of different properties extracted from these images. Note that shoulder width, hip width, and their ratio were determined from the images presented.

For the skeleton-like images, we tried to categorize the responses of the observers to the question “What helped you decide whether the image was a man or a woman?” This categorization was not an easy task, because observers were free to formulate their answer. Answers could range from a simple “no clue,” via cryptic answers such as “hips,” to an entire list of specific cues. Hence, percentages of observers mentioned in the following are not mutually exclusive. About 15% of the observers admitted that they simply had no idea what information they had been using. About 25% of the observers mentioned cues related to angles, and the most commonly mentioned were angles enclosed by markers on the torso; angles of arms; joint angles of elbows; hip joint angles or angles of the leg; mediolateral placement of feet relative to hips; and varus/valgus angles of the knee. About 15% of the observers mentioned cues related to vertical dimensions, and the most commonly mentioned were the length of the legs, the length of the upper body, and/or the length of the body as a whole. The majority of the observers, 56% in total, mentioned cues related to mediolateral dimensions. The most commonly mentioned were (the width of) the shoulders only (15%), (the width of) the hips only (8%), or (the width of) both shoulders and hips (33%); about half of the latter 33% mentioned explicitly the ratio of shoulder width to hip width. In Figure 4, for the individual SS images, we plotted the success rate as a function of shoulder width, hip width, ratio of shoulder width to hip width (all determined from the images at right heel strike), and body height. Arguably, shoulder width (Figure 4, top panel) best separated the men from the women in the SS images. Interestingly, in the observers mentioning (the width of) the shoulders only, the mean success rate in identifying the sex of SS images (77%) was higher than for the other observers (69%), and this difference was statistically significant (*Z* = 2.93; *p* = 0.003). The male with the smallest shoulder width was more often identified as a female than as a male (Figure 4, top panel). However, there was no obvious U-shape in the correct identification rate of individual images when ranked according to shoulder width (Figure 4, top panel); for example, the women with the smallest shoulder width and the man with the largest shoulder width were not identified correctly more often than others. It should be borne in mind, of course, that the order of presentation of the images was randomized for each observer. Although the observers did not know that equal numbers of male and female images were presented, the overall percentage of guesses “female” (51%) was not different from the percentage of guesses “male” (49%).

An open question is whether the observers who did not finish the questionnaire bailed out prematurely because they were bad at identifying the sex of point-light images. We have no indication that this was the case. Most of them already dropped out after seeing fewer than five images, and they had no way of knowing how they were doing because they did not receive any feedback. Interestingly, more observers dropped out in group SC–SS, which started with a block of SC images, perhaps because seeing only point clouds and guessing whether they were male or female was uninspiring; only 75% of the observers in that group reported seeing something that could be qualified as a human figure when presented with the example SC image in the block of SC images. In group SS–SC, in which the first example was an SS image, 100% of the observers reported seeing a human figure.

## Experiment 2: Sex identification on the basis of still images and movies

### Methods

#### Design, dataset, questionnaire, observer task, and statistics

**Figure 5.**
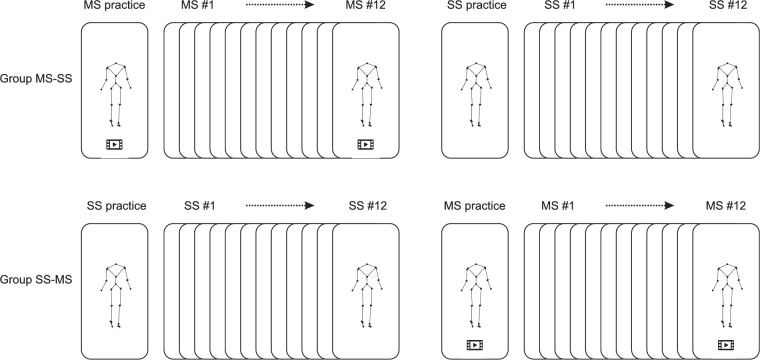
Design of Experiment 2. Each observer was randomly assigned to group SS–MS or group MS–SS. Observers in group SS–MS first saw 13 SS images and then 13 MS videos; observers in group MS–SS first saw 13 MS videos and then 13 SS images. The first image or movie in a block was for practice only.

In Experiment 2, observers again tried to identify the sex of point-light displays in the frontal plane of six walking females and six walking males. We used the same design, dataset, questionnaire, observer task, and statistics as in Experiment 1, but this time we presented the observers with frontal-plane skeleton-like still images (SS) and skeleton-like movies (MS), comprising again about half of the height of the display. SS images were selected at right heel strike, just as in Experiment 1. Each MS video was a 50-Hz real-time movie of eight walking strides, starting and ending at right heel strike. Either an SS image or an MS video was presented, and the observer indicated whether in his/her opinion the sex was male or female. Again, we used a crossover design: In random order, half of the observers were first presented with a block of still images and then with a block of movies (group SS–MS), and the other half of the observers were first presented with a block of movies and then with a block of still images (group MS–SS) (Figure 5). Just as in Experiment 1, a block consisted of 13 SS or 13 MS images, the first one of which was used only to practice the task in the block. The sex selected for the remaining 12 SS images or MS videos in the block was used for further analysis. In this experiment, we had the following specific questions:

1. Are observers more successful in identifying the sex on the basis of MS videos than on the basis of SS images?
2. Are observers more successful in identifying the sex on the basis of SS images if they have experience with identifying the sex of MS videos?

#### Observers

Observers were recruited among family, friends, acquaintances, and university students via WhatsApp, e-mail, Facebook, and Twitter. Of the 454 observers who started the questionnaire, 178 did not complete it and were excluded. Of the remaining observers, three were excluded because they answered “no” to the question of whether they had watched the images carefully. This left us with 273 observers (74 men and 199 women), 135 in group SS–MS and 138 in group MS–SS. A minority of 60 observers participated in both Experiment 1 and Experiment 2.

### Results of Experiment 2

**Figure 6.**
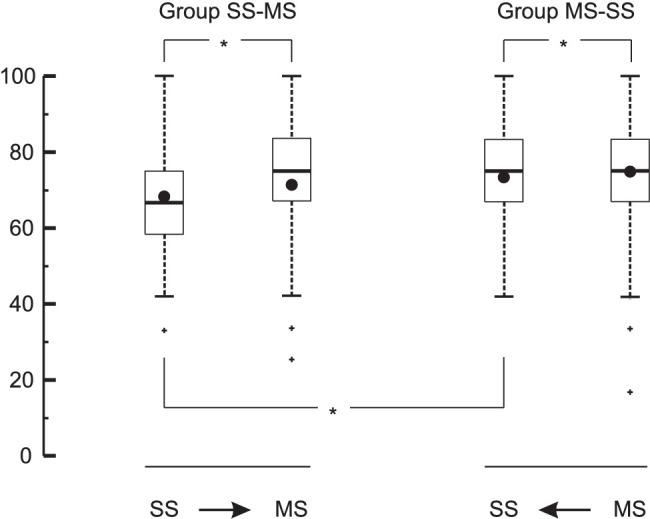
Box plots of success percentages achieved by the two groups in Experiment 2. The central horizontal line in each box indicates the median, and the bottom and top edges of the box indicate the 25th and 75th percentiles, respectively. The whiskers extend to the most extreme data points not considered outliers, and the outliers are plotted individually using plus (+) signs. The black circle in each box indicates the mean value. The arrows indicate the order of presentation for easy reference. Each asterisk (*) signals that the indicated difference was statistically significant (*p* < 0.05).

The overall mean success rate of identifying the sex on the basis of movies (73%) was higher (*Z* = 2.1600; *p* = 0.031) than that of identifying the sex on the basis of skeleton-like still images (71%). Box plots of success rates of the two groups are shown in Figure 6. In group SS–MS, the mean success rate of identifying the sex on the basis of MS videos (71%) was higher (*Z* = 2.20; *p* = 0.027) than that on the basis of SS images (68%). In group MS–SS, the mean success rate of identifying the sex on the basis of MS videos (75%) was not different (*Z* = 0.87; *p* = 0.385) from that on the basis of SS images (74%). In group MS–SS, the mean success rate of identifying the sex on the basis of SS images (74%) was higher (*Z* = 3.06; *p* = 0.002) than that in group SS–MS (68%). In principle, the 60 observers that participated in both experiments could have transferred something they learned in one experiment to the other experiment. However, reassuringly, in group SS–MS the mean success rate of identifying the sex on the basis of SS (68%) was not different (*Z* = 1.34; *p* = 0.18) from that in group SS–SC in Experiment 1 (70%); in both cases, the same SS images were shown in the first block.

For both the images and the videos we tried to categorize the responses of the observers to the question “What helped you decide whether the image was a man or a woman?” For SS images, 80% of the observers mentioned one or more of the cues that were reported in Experiment 1. For MS videos, 88% of the observers mentioned dynamic cues; hip movement was mentioned by 52% of the observers, arm movement by 20%, foot movement by 14%, shoulder movement by 12%, torso movement by 6%, and “swing” of one or more of these body parts was explicitly mentioned by 11%. Interestingly, only 25% of the observers mentioned any static cue for deciding on the sex on the basis of MS videos.

## Discussion

In this study, we set out to determine to what extent observers need dynamic information to identify the sex of a walking person in the frontal plane. We first studied the success rate in sex identification on the basis of point-light still images, which by definition do not contain dynamic information. In Experiment 1, we showed that the success rate on the basis of the frontal-plane still images that we used was above chance. Hence, our first conclusion is that observers are able to extract sex-related information from point-light still images. Our observers had a higher success rate on the basis of skeleton-like still images than on the basis of cloud-like still images; the median success rate on the basis of skeleton-like still images was as high as 75% (Figure 3), which means that the majority of the observers correctly identified the sex of at least nine out of 12 images without using any dynamic information. In Experiment 2, we showed that the success rate was not necessarily higher on the basis of skeleton-like movies than on the basis of skeleton-like still images; observers in group MS–SS performed just as well on the basis of still images as they did on the basis of movies (Figure 6). Hence, our second conclusion is that dynamic information plays at most a secondary role in sex identification in the frontal plane. Below, we discuss our findings in the context of the literature in the field and address the question of why our conclusions are different from those of previous studies.

Davis and Gao (2004) were the first to study the success rate of observers in identifying the sex of frontal-plane cloud-like still images. The success rate of their 15 observers was 57%, which was statistically significantly above chance. We used in our study similar cloud-like still images, and our observers achieved on average a success rate of 63%, also statistically significantly above chance (*p* < 0.001). It seems, therefore, that human observers can identify the sex of frontal-plane still images with a success rate above chance. This seems opposite to the results of Kozlowski and Cutting (1977), who concluded that, for sagittal-plane cloud-like still images, “Recognition of the sex of a walker is simply not possible from four static arrays taken from the dynamic sequence.” However, it may be argued that most of the anthropometric differences between men and women are directly visible in the frontal plane and not in the sagittal plane (Cutting et al., 1978). Because the success rate of sex identification may depend on whether sagittal-plane or frontal-plane images are used, our conclusion does not necessarily conflict with that of Kozlowski and Cutting (1977).

In our study, observers identified the sex not only on the basis of cloud-like still images but also on the basis of skeleton-like still images. Overall, our observers had on average a 7% higher (*p* < 0.001) success rate in identifying the sex on the basis of SS images than on the basis of SC images (Figure 3). It may be concluded, therefore, that connecting the marker dots with lines to represent body segments enhanced the success rate of observers in sex identification. It seems that the sex-related information can be extracted more successfully when lines are added to help the observer understand what the point lights represent and to see the actual body segments, rather than having to imagine them.

**Figure 7.**
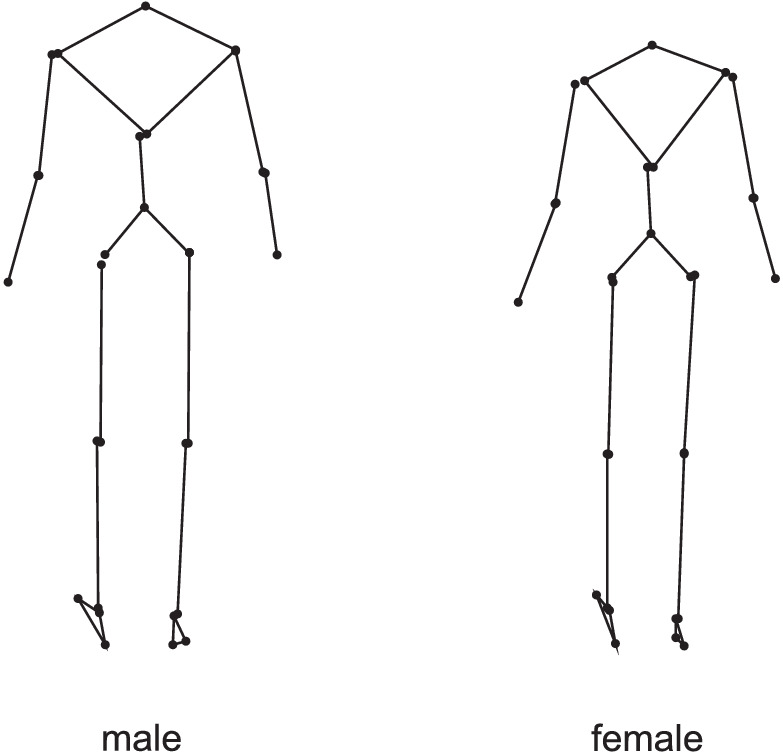
Average skeleton-like point-light images of the male (*n* = 6) and female (*n* = 6) walkers at the instant that the right heel contacted the ground.

What information could observers be extracting from frontal-plane still images? In principle, a snapshot from a continuous motion may happen to be taken at the instant that a maximum excursion of a joint is reached, and this maximum excursion may, on average, be different for males than females. For example, the still image could give a clue as to the amplitude of lateral body sway in shoulders and hips in males and females. However, we see no sign of that in Figure 7, which shows the average male and female still images used in our study. What we do see in Figure 7 is that, even though at the instant of right heel strike a human walker is not in the standard anatomical position (i.e., standing erect and at rest with the feet together), our images contain a lot of anthropometric information. Most obviously, the images of the males were simply bigger on average than the images of the females. In the course of observing a series of images one by one, observers could work out a running average size and classify all images larger than that as male and all images smaller than that as female. In the images that we presented to the observers, this strategy to distinguish men from women would be more successful using shoulder width as cue than using hip width or body height as cues (Figure 4). It is of note, therefore, that observers who reported using shoulder width had higher (*p* = 0.003) mean success rates in identifying the sex of SS (77%) than observers who reported using cues other than shoulder width (69%). We chose to preserve size information in the images because we felt that this was ecologically valid. After all, body size is part of the structural information that can be used for sex identification in real life. However, one can also choose to scale the images to a fixed height so that only proportional differences between men and women remain, as was done by Davis and Gao (2004). This difference in choice could explain why our observers achieved on average a 6% higher success rate in identifying the sex of SC images than observers in the study by Davis and Gao (2004). When it comes to proportional differences, it has been suggested in the literature that the ratio of shoulder width to hip width is an important sex clue. And, in fact, 33% of the observers reported using (the width of) both shoulders and hips as cues when identifying the sex of SS images, and half of these observers mentioned specifically the width of the shoulders relative to the width of the hips as a cue. This is in line with Saunders, Williamson, and Troje (2010), who studied eye movements of observers and concluded that, for the perception of sex, the shoulders and hips are particularly important. In the images that we used in our study, however, there was considerable overlap between the men and women in the ratio of shoulder width to hip width (Figure 4), rendering this cue less reliable. In general, this indicates that in studies on sex identification of point-light walkers in which kinematic data of only a few men and women are used, the reliability of cues and the absolute success scores of observers will also depend on the particular walking subjects selected and therewith the overlap in absolute dimensions and proportions between the men and the women.

When it comes to identifying the sex of point-like walkers, the general opinion in the field is that observers rely heavily on dynamic information (i.e., information on the motion pattern). However, in two key studies in which this was concluded for the frontal plane (Mather & Murdoch, 1994; Troje, 2002), there was no direct comparison between still images and movies; rather, the conclusion was based on the success rate of observers in identifying the sex of avatars on the basis of movies, in which the anthropometry, posture, and dynamic gait characteristics of the avatars had been manipulated. Possibly, the manipulation caused the avatars to be different from real walking people. Davis and Gao (2004) did make a direct comparison between frontal-plane still images and movies, and they found that the success rate of observers in sex identification was higher on the basis of movies (69%) than on the basis of still images (57%). They concluded that “… dynamic stimuli appear to give more gender-related information than the single frame case.” However, the authors used cloud-like images and movies, and the results of our Experiment 1 suggest that it is important for the observer to know what the point lights in an image represent. Does motion still have added value when lines are added to clarify what the point lights represent, as in SS images? In Experiment 2, we found that the observers in group SS–MS did indeed have a higher (*p* = 0.027) mean success rate on the basis of movies (71%) than that on the basis of still images (68%) (Figure 5). The observers in group MS–SS had an even higher mean success rate on the basis of still images (74%) and performed just as well (*p* > 0.05) as on the basis of movies (75%).

How can we explain why group MS–SS had a higher success rate on the basis of still images than group SS–MS? The only explanation we can think of is that first seeing a block of movies helps naïve observers understand that the still images represent people that are walking. When they understand this and are experienced (Reid, Brooks, Blair, & van der Zwan, 2009), then motion information is no longer needed for sex identification. Hence, our second conclusion is that dynamic information plays at most a secondary role in sex identification of walking persons in the frontal plane. When it comes to identifying the sex on the basis of point-light still images, this conclusion holds under the condition that the observer is able to perceptually group the point lights or segment them to represent a human body that is frozen at heel strike during a walking cycle. If the point lights are presented in a cloud, then this grouping may require seeing them in motion (Casile & Giese, 2005; Garcia & Grossman, 2008; Johansson, 1973; Lu, 2010; Mather, Radford, & West, 1992; Troje, 2002) or at different points and hence different configurations in the walking cycle (Beintema & Lappe, 2002; Giese & Poggio, 2003; Lange & Lappe, 2006; Lappe, Wittinghofer, & de Lussanet, 2015). Sex identification can then perhaps be based on matching the mentally created structural information to learned male and female body templates. In the study of sex identification on the basis of point-light images, this whole process is facilitated by showing lines representing the body segments, so that the observer does not have to imagine them, and by knowing that the body is frozen at a particular point during a walking cycle. In life, however, the full walking human body is directly visible, including body contours, and dynamic information may not be needed for sex identification.

The responses of the observers to the question “What helped you decide whether the movie was a man or a woman?” seem to contradict our second conclusion, that dynamic information plays at most a secondary role in sex identification of walking persons in the frontal plane. After all, 88% of the observers mentioned using dynamic cues for deciding on the sex on the basis of movies, and only 25% of the observers mentioned any static cue (down from 80% mentioning static cues in the case of still images). Why would the observers not rely on the static cues, such as body size, which are obviously still available in a movie and sufficient to achieve a success rate as high as 74%? Are the observers truly switching from static cues to dynamic cues when deciding on the sex on the basis of movies, only to end up at the same success rate? Or are they merely *reporting* the use of dynamic cues while subconsciously still using the static cues? Needless to say, we cannot answers these questions using the data that we have collected. In any case, showing a full movie rather than a single skeleton-like still image did not lead to higher success rates in sex identification in the frontal plane.
